# Gaps in access to essential medicines and health products for noncommunicable diseases and mental health conditions

**DOI:** 10.2471/BLT.20.272658

**Published:** 2020-09-01

**Authors:** Ren Minghui, Mariangela Simao, Bente Mikkelsen, Dévora Kestel, Andrew Ball, Zsofia Szilagyi

**Affiliations:** a Division of Universal Health Coverage, Communicable and Noncommunicable Diseases, World Health Organization, avenue Appia 20, 1211 Geneva 27, Switzerland.; b Division of Access to Medicines and Health Products, World Health Organization, Geneva, Switzerland.

In 2019, heads of state pledged to strengthen efforts to address noncommunicable diseases and mental health as part of universal health coverage. They committed to progressively cover 1 billion additional people within four years with essential health services, as well as quality, safe, effective and affordable essential medicines and other health products.^1^ The coronavirus disease 2019 (COVID-19) pandemic has slowed these efforts and highlighted the vulnerability of people living with noncommunicable diseases and mental, neurological and substance use disorders,^2^ referred to as mental health conditions.

Once the COVID-19 emergency is over, governments will need to take a critical look at their policies with regard to noncommunicable diseases and mental health conditions. Governments need to learn lessons from this crisis and use them to catalyse, reconceptualize and rebuild the global response to noncommunicable diseases and mental health. The response needs to be comprehensive and based on implementing the World Health Organization’s (WHO) core technical guidance in these areas, including the most cost–effective noncommunicable disease interventions,^3^ or best buys.

The pandemic has put a spotlight on the need to strengthen health infrastructures (with primary health care at their core), build better data systems, tackle the global shortage of health workers, and address the drivers of the global treatment gap for noncommunicable diseases and mental health conditions. As the health community rethinks the global noncommunicable disease and mental health response, these system failures will need to be comprehensively addressed.

Here we focus on access to essential medicines and other health products, which we believe needs to become a priority for governments. To take forward the access agenda, the *Roadmap for access to medicines, vaccines and health products (2019–2023)*^4^ will guide WHO’s efforts. WHO has also drawn key lessons from the global response to high-mortality communicable diseases, and will apply them in the context of noncommunicable diseases and mental health.

We have identified five key lessons and five overarching developments that will be critical to expanding access to medicines and health products for noncommunicable diseases and mental health conditions, and where WHO can provide additional guidance.

The first is development of robust evidence-based normative guidance, which, in the context of communicable diseases, has proved to be a key resource for countries and development partners. This guidance is also an incentive for pharmaceutical and other manufacturers, helping to rapidly increase access to innovative new health products.

The second is the role of WHO’s Prequalification Programme and WHO’s *Model list of essential medicines*^5^ and the *Model list of essential in vitro diagnostics*.^6^ In November 2019, WHO launched its first-ever insulin prequalification programme,^7^ which will increase choice, reduce prices and expand access to diabetes treatment in low- and middle-income countries and can be replicated for other conditions.

The third is our work on affordability and fair pricing, which has involved increasing transparency of pricing approaches and strengthening price negotiation capacities at the country level. This work was expanded to noncommunicable diseases in 2018, when WHO looked into pricing approaches in relation to cancer medicines and their impact on availability and affordability.^8^

The fourth area is demand forecasting for diagnostics, medicines and vaccines to prevent shortages. Having estimates of global demand for active pharmaceutical ingredients, medicine formulations and diagnostics has had a crucial impact on shaping manufacturing trends, and could bring similar benefits for the noncommunicable disease and mental health fields.

The fifth area is a greater push for pooled procurement, which could include a global drug purchase facility. WHO has initiated a multisectoral dialogue to assess the feasibility of a global drug facility for essential noncommunicable disease medicines, building on the successes of similar platforms for communicable diseases and vaccines.

We suggest that these efforts would be further amplified through advances in associated areas, including: (i) establishing grassroots civil society organizations for noncommunicable diseases and mental health that create demand and push for rights-based approaches and accountability; (ii) strengthening national financing mechanisms to promote long-term and sustainable funding; (iii) creating a global accountability mechanism for monitoring access to all essential medicines,^9^ (iv) increasing attention to non-pharmacological approaches in disease management and control, and (v) leveraging broader partnerships, including public–private partnerships.

All these efforts will be needed post COVID-19, to reboot and rebuild efforts around noncommunicable diseases and mental health. Without increased attention to these priority areas, integrating interventions in these areas into the full continuum of care, and moving closer to relevant targets of the sustainable development goals, will be challenging.
